# Low to medium-low risk perception for dengue, chikungunya and Zika outbreaks by infectious diseases physicians in France, Western Europe

**DOI:** 10.1186/s12889-019-7317-9

**Published:** 2019-07-31

**Authors:** Marion Le Tyrant, Daniel Bley, Catherine Leport, Serge Alfandari, Jean-François Guégan

**Affiliations:** 1 UMR ESPACE 7300, CNRS, Aix Marseille University, Avignon Université, Université Nice Sophia-Antipolis, F-13545 Aix-en-Provence, France; 2 UMR MIVEGEC, IRD, CNRS, University of Montpellier, Centre IRD de Montpellier, F-34394 Montpellier, Cedex 5 France; 3 Université Paris-Diderot, Inserm 1137, UMR 1137, 16, rue Henri-Huchard, 75870 Paris, Cedex 18 France; 4 Mission COREB Nationale, Assistance publique – Hôpitaux de Paris, 75004 Paris, France; 5 Service de réanimation et maladies infectieuses, Centre hospitalier de Tourcoing, Tourcoing, France; 6 UMR ASTRE, INRA, Cirad, University of Montpellier, Campus international de Baillarguet, Montferrier-sur-Lez, F-34980 Montpellier, France; 7 International U.N. programme FutureEarth, OneHealth global research programme, Montréal, Canada

**Keywords:** Chikungunya, Dengue, Zika, Infectious diseases physicians, Risk perception, Vector-borne disease, Western Europe

## Abstract

**Background:**

Many tropical countries are currently experiencing dengue (DEN), chikungunya (CHIK) and also more recently Zika (ZIKA) epidemics (particularly in Latin America). Although the risk of transmission and spread of these infections in temperate regions remains a controversial issue, vector-borne diseases have been widely reported in the media and have been the focus of preventive strategies by national and international policy-makers and public health authorities. In this context, we wanted to determine the extent of risk perception in infectious diseases (ID) physicians of the current and future risk of arboviral disease introduction, autochthonous case development and epidemic scenarios in France, Western Europe.

**Methods:**

To this aim, we developed an original standardized questionnaire survey which was disseminated by the French Infectious Diseases Society to ID physician members.

**Results:**

We found that ID physicians perceived the risk of introduction and outbreak development of DEN, CHIK and ZIKA in France to be low to medium-low. Generalized Linear Model(s) identified medical school training, the extent of professional experience, and awareness of the French national plan regarding arboviral infections as significant predictors for lower risk perception among respondents.

**Conclusion:**

Despite the fact that arboviral diseases are increasingly being imported into France, sometimes resulting in sporadic autochtonous transmission, French ID physicians do not perceive the risk as high. Better communication and education targeting health professionals and citizens will be needed to enhance the effectiveness of the French national plan to prepare against arboviral diseases.

**Electronic supplementary material:**

The online version of this article (10.1186/s12889-019-7317-9) contains supplementary material, which is available to authorized users.

## Background

The progressive establishment of *Aedes albopictus*, the tiger mosquito, in southern Europe at the beginning of the 90’s and its establishment in France in about 2004 have increased the potential health threat posed by these arthropods in temperate countries [1]. Many tropical arboviral diseases transmitted by arthropod vectors, such as dengue fever (DEN hereafter), chikungunya (CHIK) and Zika (ZIKA) transmitted by *Aedes* mosquitoes, are now also seen as emerging threats in temperate and sub-temperate regions. The Mediterranean basin, which offers suitable environmental conditions for mosquitoes, is considered to be at high risk for outbreaks of new arboviral diseases [2]. Since 2010, sporadic cases and small outbreaks of DEN and CHIK viruses have been recorded in Western and Southern Europe [3–8] A small outbreak of DEN with 15 cases took place in Croatia in 2010 [9] followed by a much larger epidemic on the Portuguese Island of Madeira in 2012 [10] with over 2,000 human cases. Autochtonous DEN transmission has been repeatedly reported in France as well since 2010 [11–13]. To our knowledge, no autochthonous case of ZIKA has been declared on the sub-continent, despite sporadic sexual transmission of the virus reported in France [14], Germany [15] and Italy [16]. Between early August and mid-September 2017, 17 autochtonous CHIK cases (15 confirmed and 2 probable) have been reported in two cities of the Var department, in the southeast of France. The primary case was imported via a return from Cameroon, in central Africa. This 2017 outbreak was the ninth episode of local autochtonous CHIK virus transmission in mainland France [17]. After this outbreak the French national public health agency “Santé publique France” in charge of human health highlighted the need for public awareness and training campaigns targeting healthcare professionals.

Santé publique France regularly publishes information concerning, notably, these three infections. As a recent illustration, from May 1st to June 7th of 2019, 109 DEN, 12 CHIK and 1 ZIKA confirmed imported cases were reported in mainland France, with 40% of dengue cases originating from Réunion Island where a major DEN epidemic has been ongoing since the beginning of 2019. During the same period, no autochtone cases of DEN, CHIK and ZIKA were reported by this national health agency in mainland France [18]. Although importation to Europe via travelers is well documented, the true risk of establishment of these three arboviral diseases after importation remains unknown. The risk of disease introduction into mainland France and Western Europe is exemplified by the massive flow of air transportation to and from tropical regions, notably to and from ultraperipherical regions, and by its increase through the years: in 2018 the number of passengers entering mainland France from these areas included 2,475,116 from Réunion Island (increase rate of 7,9% from 2017 to 2018), 2,446,234 from Guadeloupe (3,6%), 1,978,356 (2,4%), 1,393,849 from Tahiti (7,9%) and 538,782 from French Guiana (4,7%) among others [19].

The first mathematical modeling study for the risk of DEN virus establishment in Europe was published recently [20], and actually showed the risk to be low. However, climate change will increase the risk of arboviral diseases as the seasonal window for suitable temperature conditions for the settlement of *Aedes* mosquitoes and viral transmission increases in Europe, and especially in Southern Europe [21]. Furthermore, the number of travelers from DEN endemic and CHIK or ZIKA epidemic countries to Europe is increasing at an unprecedented rate. According to [20], the highest number of DEN virus importations via air travelers are projected to occur in Germany, France and the United Kingdom, with both France and Italy which have significant presence of *Ae. albopictus* that should know an important number of modeled dengue infected air passengers [20].

Major epidemics have happened in Réunion Island during the last 15 years: 2005–2006 with 244,000 cases of CHIK (near 40% of the population) and 203 deaths; a series of DEN outbreaks in Guadeloupe, Martinique and French Guiana, with an increase in severe forms, particularly dengue haemorrhagic fever [22]; in Réunion Island, the epidemiology of DEN is moving from an endemo-epidemic situation towards a hyper-endemic situation, and it may affect up to 5% of the population. The epidemiological dynamics observed over this period raise fears of a move towards a situation comparable to that currently seen in Southeast Asia. DEN could become one of the leading causes of hospitalization, especially for children. For instance, during the two 2005 and 2007 DEN epidemics in Guadeloupe (400,500 inhabitants in 2007), the number of clinical cases that led to a medical consultation were respectively 11,500 in 2005 (0.4% of severe cases; serotype 4 predominant) and 19,000 in 2007 (0.8% of severe cases; serotype 2 predominant); in 2018, the number of DEN cases in Guadeloupe and Martinique also raised the epidemic threshold. In December 2013, the first autochthonous cases of CHIK in the Americas were recorded in the French-Dutch Caribbean island of Saint-Martin. The virus spread to other nearby islands of the French West Indies (Saint-Barthélemy, Martinique and Guadeloupe), to the majority of Caribbean islands and to continental America. This epidemic has probably involved more than one million people; in 2014, at least 81,200 presumed clinical cases of CHIK fever were recorded in Guadeloupe, and 72,500 in Martinique [23]. In Réunion Island again, after the 2017 outbreak of DEN disease, near 8000 cases have been estimated from the beginning of 2018 until the present; concerning the ZIKA epidemic between June 2015 and March 2017, 1141 cases have been reported in French overseas departments, i.e., Guadeloupe (489 cases), Martinique (421) and French Guiana (231).

Faced with public concern and widespread media coverage, national health authorities and policy-makers reacted by implementing national and global health measures to fight these new infections [24]. In France including ultraperipherical territories, a national plan against the spread (NPS) of dengue, chikungunya and Zika was implemented in 2006 and is updated each year to prevent the expansion of *Ae. albopictus* in mainland France, and to organize the surveillance of human cases. Moreover, the French medical and research communities have rapidly developed interdisciplinary programs to better understand and fight these new diseases, for instance the Research and ACTion targeting emerging infectious diseases (REACTing) [25]. From 2009 to 2016, the general French population’s awareness of these arboviral risks has strongly changed; from a low awareness among the population [26], citizen views of potential risks has increased during this period with some heterogeneities observed depending on the region and mosquitoe settlement [27]. Meanwhile, French national health authorities have continuously pursued information campaigns on the potential risks of transmission of these three arboviral infections.

Despite this national effort, no one has tried to quantify the estimation and perception in the different categories of health professionals who are in contact with infected patients of the current and future risk of arboviral diseases. Here, we focused on infectious diseases (ID) physicians because they are well-trained to cope with new emerging infectious threats and also to deliver an objective expert assessment of the real risks of new infections. Moreover, they understand the complexity of vector-borne disease appearance and propagation outside their traditional endemic areas. Our main objective was to evaluate, using an online questionnaire and through a cross-sectional study, their perception of the current and future (10-year) risks of introduction, sporadic case occurence and epidemics of DEN, CHIK and ZIKA in mainland France. We then analyzed the influence of geographic or environmental variables (e.g., presence of insect vectors) and infrastructures (e.g., international airports), as well as that of medical training (e.g., medical school and continuing education), and NPS awareness, on risk perception in ID specialists. Our initial hypothesis was that mosquito biology and international transportation facilities should more significantly influence the perception of a potential threat in this category of health professionals. Therefore, professional respondents’ views of risk perception for potential infections among the general population should be higher among ID physicians located in such departments than in any other departments.

## Methods

### Data collection and participants

For this study, the French Infectious Diseases Society (SPILF) kindly helped us by sending to the 685 hospital physicians registered on their “Infectio-flash” Discussion List a questionnaire we developed on their perception of the current and future (10-year) risk of introduction, sporadic cases and epidemics for DEN, CHIK and ZIKA, three important vector-borne diseases that are transmitted to humans by two species of mosquitoes (*Ae. aegypti* and *Ae. albopictus*) [28]. We only considered mainland France, and excluded all French overseas territories in order to focus on the risk of disease emergence in mainland France, where *Ae. albopictus* has settled during the last couple of decades, and *Ae. aegypti* is absent. The presence of *Ae. albopictus* in France is monitored at the departmental level [8]. We selected these three diseases due to: i) the strong human transportation connections between mainland France and its tropical overseas territories: French Guiana and French West Indies (DEN, CHIK and ZIKA), Reunion Island (DEN, CHIK), and French Polynesia (DEN, CHIK and ZIKA); ii) the huge numbers of international tourists visiting France each year (up to 83 million visitors in 2016); and iii) the social, economic and political impacts of the CHIK epidemics on Reunion Island in 2005–2006, and in Emilia-Romagna, an Italian region close to southeastern France. All these conditions render certain regions of mainland France potentially vulnerable to these new emerging diseases.

The questionnaire (see Additional file 1: Appendix I) was uploaded on a dedicated Google Forms website and the link was sent to all ID physicians registered with SPILF. Members were informed of this scientific investigation and its issues, were totally free to reply to it, or not, and when replying to the online questionnaire consented to the terms and conditions of this study. The questionnaire was completely anonymous, and practitioners were referenced with a personalized digital code. We did not collect sensitive data, in accordance with current ethics rules (see at: https://ethiquedroit.hypotheses.org/1717#more-1717; see also article 89 from the European rules, April 27th 2016 and article 40.II January 6th 1978).

The sample population (see Additional file 1: Appendix II for further details) was 47,5 years old on average (47 years old for the overall SPILF population, as of 2018], with a median age for MD thesis dissertation being 31 years old (30 years old), a sex ratio of 61/39 (male/female) (48/52), and training in infectioliogy and duration of internship of 1 year; 4 to 5 years of medical specialization plus 1 year of post-internship (for people trained between 1984 and 2017 which included all respondents to the questionnaire (from 2018 to the present, training is only 5 years and infectiologists receive an educational degree called a DES diploma).

Pre-versions of the questionnaire were sent to different public health authorities and medical staff members (regional public health agency - Occitanie, Santé publique France, welfare system - Paris Hospitals, regional hospitals...) in order to improve questions’ accuracy and intelligibility. Even if our questionnaire was not pre-tested on a subset of participants, its validity and reliability were determined according to feedback exchanges on improvements to the questionnaire with these different public health and medical personnel.

The questionnaire included 58 main questions, some of which (e.g., “Today, how do you evaluate the epidemic risk of DEN, CHIK and ZIKA in mainland France?”) were divided into three sub-sections to separately analyze the three infectious diseases. Finally, the questionnaire included 72 (sub-)questions (and thus, variables). Moreover, six additional variables were extracted a posteriori from the information included in the completed questionnaires: latitude and longitude (in degrees, minutes and seconds transformed in decimal degrees) of the respondent’s workplace, presence of an international airport in the respondent’s department (coded 1/0), *Ae. albopictus* presence (coded 1/0), population size of the city registered as the respondent’s workplace (number of inhabitants), and registered autochthonous cases of DEN and CHIK (number of cases) in the respondent’s department. A French department is an administrative territory, and mainland France includes 96 departments.

Questions and sub-questions were grouped into eight different categories: i) estimation of the total number (current and future) of imported DEN, CHIK and ZIKA cases, in the department and nationwide; ii) perception of sporadic autochthonous case development of DEN, CHIK and ZIKA (current and future) in the department and nationwide; iii) global perception of autochthonous epidemic events of DEN, CHIK and ZIKA (current and future) in the department and nationwide; iv) estimation of the level of concern about the risk of sporadic DEN, CHIK and ZIKA cases (current and future) in the department and nationwide; v) estimation of the level of concern about the global (all three diseases together) risk of epidemic events (current and future) in the department and nationwide; vi) perception of the severity of the clinical consequences (symptoms, complications, mortality…) of DEN, CHIK and ZIKA epidemics; vii) perception of the socio-economic impact of DEN, CHIK and ZIKA; and, viii) qualitative estimation of the communication by public health authorities on DEN, CHIK and ZIKA.

### Statistical analysis

All the estimations were rated on a 10-point Likert scale [29] with 0 being the lowest and 10 the highest level. Reliability of the study instrument was determined using Cronbach’s α. Because we have several questions that are heavily dependent on some core questions, we calculated Cronbach’s α by two different means. Cronbach’s α taking into account all questions yielded a value of 0.938 (number of items is 72) and Cronbach’s α using only main questions yielded a value of 0.789 (number of items is 58), suggesting that the items in our questionnaire have relatively high internal consistency.

First, univariate regression models were used to investigate the relationships between the current and future risk as perceived by hospital practitioners and the different independent variables extracted from the questionnaire (see Table 1), and this for all three diseases. Second, scatter diagrams were used to visualize the plot distribution between current (x-axis) and future (y-axis) disease risk perception for the three infectious diseases. The locally-weighted scatterplot smoothing (LOWESS) non-parametric regression method was used to characterize the main perception trend. A flat plot distribution indicates the perception of a future low/absent disease risk; conversely, a plot distribution near or above the x = y line indicates a future risk perception equivalent to or higher than the current one. Non-parametric and parametric tests were used, when adequate, to evaluate correlations between responses and explanatory variables [30].

**Table 1:** Current and future estimation (on a 10-point Likert scale) of the different disease scenarios for dengue (DEN), chikungunya (CHIK) and Zika (ZIKA) (imported or autochthonous cases, and epidemics) within the respondents’ department and nation-wide

Arboviral disease scenarios		Mean	S.D. (±)
Imported cases today	of DEN, in France	2.570	1.558
	of CHIK, in France	2.253	1.523
	of DEN, in the department	1.987	1.532
	of CHIK, in the department	1.705	1.504
Imported cases in 10 years	of DEN, in France	3.949	1.844
	of CHIK, in France	3.700	1.796
	of DEN, in the department	3.256	1.964
	of CHIK, in the department	3.077	1.871
Autochthonous cases today	of DEN, in France	3.734	2.123
	of CHIK, in France	3.696	2.162
	of ZIKA, in France	3.583	2.187
	of DEN, in the department	2.456	2.246
	of CHIK, in the department	2.513	2.328
	of ZIKA, in the department	2.389	2.236
Autochthonous cases in 10 years	of all, in France	4.472	2.611
	of all, in the department	3.364	2.486
Epidemic scenario today	of DEN, in France	1.987	1.791
	of CHIK, in France	1.974	1.721
	of ZIKA, in France	1.959	1.670
	of DEN, in the department	1.597	1.858
	of CHIK, in the department	1.688	1.907
	of ZIKA, in the department	1.458	1.694
Epidemic scenario in 10 years	of all, in France	3.234	2.212
	of all, in the department	2.556	2.391

The relatively small sample size of respondents prevented using many multivariate analyses. However, general linear (GLM) and Generalized Linear Model(s) were used to analyze the influence of the different explanatory variables, and tentatively their two-way interaction terms, on the perception of future risk by developing null and minimal models [31]. In the GLMM models, variables, such as age and date of medical degree, were used as random variables, and other variables were used as fixed factors. As we did not want to produce the best-fitted explanatory models for the future disease risk perception, the dependent and independent variables were kept untransformed in multivariate models. However, the normality of distribution and homoscedasticity were checked with the Shapiro’s test. To relate the future risk perception variables to independent factors, a Gaussian and a Poisson error model were used, and factors and their interaction terms were selected by using a backward-forward stepwise elimination procedure from the general models and according to the Akaike Information Criterion (AIC) [31]. Variables were selected using the analysis of variance (ANOVA), with tests specified as “type-III” to assess the effect of each variable after accounting for all other factors [32].

The robustness of our results relative to sampling heterogeneities was tested using a modified rarefaction analysis. Random samples were generated that contained from 40 to 100% of all questionnaire data for each of the three arboviral diseases. The random sampling was repeated 10 times, and the primary analysis was run using each of these random samples. This allowed us to test the robustness of each result and exclude findings that were significant only due to the presence of outliers.

All analyses were performed using Systat ver. 13.1 (Systat Software Inc., CA) and S-Plus 4.5 (TIBCO Software Inc., CA).

## Results

### Participation rate and disease scenarios

The questionnaire was accessible on line between January and May 2016, and we received 80 replies (11.7% of 685). Among the 80 respondents, 33 replied to all questions, 27 omitted between 1 and 3 questions, 8 did not reply to between 4 and 6 questions, and 12 did not reply to ≥7 questions (mean number of omitted questions ± SD = 4.687 ± 9.832). This response rate was considered to be normal for this type of questionnaire, although not totally satisfactory for the category of interviewed professionals.

Their estimation of the current and future (10-year span) imported and autochthonous cases and epidemics nationwide and within their department are described in Table 1.

### General trends for the perception of future disease risk

**Fig. 1:**
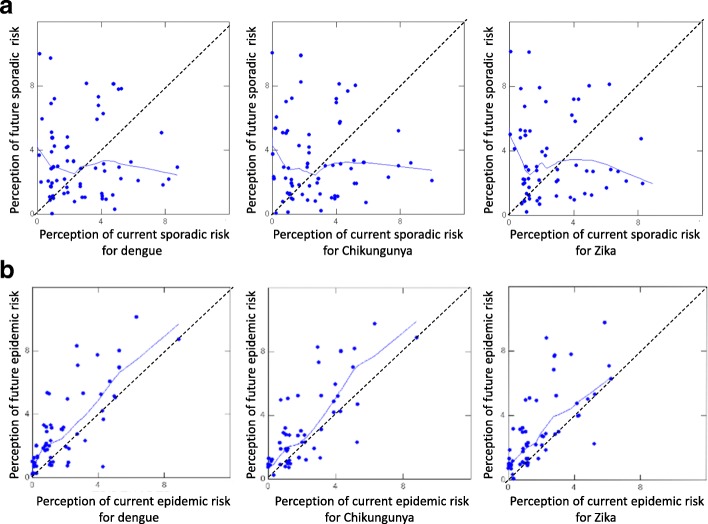
Relationships between (**a**) the perception of the future (*y*-axis) and current (*x*-axis) risk of sporadic cases, and (**b**) of epidemics for dengue (DEN), chikungunya (CHIK) and Zika (ZIKA) at the department scale (similar results were obtained at the national scale). **a** shows a high dispersion of *y*-axis responses for *x*-axis values lower than 4, and a tendency towards more homogeneous *y*-axis responses (low to medium-low scores) with increasing *x*-values (see text for further details). The dotted line indicates identical risk perception values for today and the future (*x* = *y*). The blue line corresponds to the locally-weighted non-parametric curve that gives the main trend

**Fig. 2:**
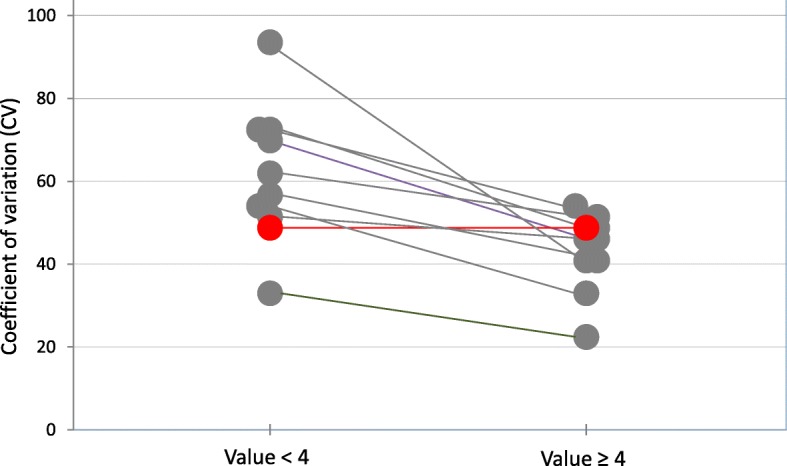
Coefficient of variation (CV) values allowed the categorization of the perception of future and current risk in two classes based on a cut-off of 4 (< 4 and ≥ 4) that described the main trend for the different situations and different arboviral diseases. Generally (dots in grey color), the coefficients of variation (CV) values were higher for values lower than 4 (corrected *CV* = range values between 36.160 and 93.415, *p <* 0.001) than for values higher or equal to 4 (corrected *CV* = range values between 22.004 and 50.095, *p <* 0.001). Conversely, for the imported cases of dengue nation-wide (in red color), the variation coefficients were identical in both groups (corrected *CV* = 43.553 and 44.420, respectively, *p =* 0.995). See text for further details

By plotting the current disease risk perception (x-axis) against the future disease risk perception (y-axis) (Fig. 1), we found, for all three diseases, a flat relationship for the sporadic autochthonous cases both at the departmental and national levels (Fig. 1a). By using linear parametric or LOWESS non-parametric regressions, the perception of future disease risk by ID physicians was always flat, indicating no trend for an increasing disease risk over time (Fig. 1a). Nevertheless, we observed a broad dispersion of the responses for the perception of future risks, with a higher dispersion when the current risk perception values were lower than 4 compared with values higher or equal to 4, except for imported cases of dengue at the national level (see Fig. 2 for further details). With higher levels of current risk perception, the future risk perception responses tended to become more homogeneous towards low to medium-low scores. Conversely, the perception values of the future global disease epidemic risk (all three diseases together; y-axis), both within the department and nationwide (Fig. 1b), tended to increase with the increase of the present risk perception values, with curves that more or less followed the x = y diagonal line. This last finding indicates that ID specialists are aware of the risk of epidemic appearance, but they are at the same time unable to qualify the type of risk, e.g., which specific category of arboviral diseases will spread.

### Main determinants of disease risk perception: importance of professional training and health communication

For clarity, and because our study generated many different statistical analyses and results, we decided to summarize the main findings in Table 2, and to focus on some specific results. Table 2 lists the main explanatory variables retained in both null and minimal linear models, based on their coefficient values, for the three distinct scenarios of arboviral disease appearance and propagation (from imported cases to autochthonous cases, and finally epidemic state for DEN, CHIK, and ZIKA), at the department and national levels. Over all, we observed that training activities and NPS awareness by respondents were, in most situations, the most important parameters by which to explain the different gradations of disease risk perception. The only exception was the perception of autochthonous case scenarios within the respondent’s department for which the presence of an international airport, the presence of tiger mosquitoes, and the identification of existing autochthonous cases were the best explanatory variables. For the epidemic state situation, longitude was an additional explanatory variable at both department and national levels. This is explained by the respondent’s distance from oceanic or Mediterranean regions that are more favorable for the establishment of a tiger mosquito population.

**Table 2:** Main significant predictor variables at 5% for dengue, chikungunya and Zika risk perception (imported or autochthonous cases, epidemic scenario), at the department and national scales. Summary results for both current and future disease risk perceptions by ID physicians. Underlined variables correspond to training activities by the respondents, and variables in normal characters to geographical, environmental or epidemiological variables. The scenarios in boxes (1, 2 and 3) are described in the main text

Risk gradient			
	Imported	Autochthonous	Epidemic
Departmental scale	Scenario 1 Birth year, Specialty Birth year × Infect. spe., Birth year × NPS	Scenario 2 International airport, *Ae. albopictus* presence, Identification of autoch. Cases, NPS	Year of medical degree, Longitude
National scale	Birth year, Year of medical degree, NPS	Additional training spe., Tropical experience	Scenario 3 Year of medical degree,. Tropical experience, Additional training spe., Longitude

#### Introduced cases situation

For the scenario of disease case introduction, we only obtained significant explanatory linear models for DEN, but not for CHIK and ZIKA (for the perception of the future risk of chikungunya case introduction nationwide, only the NPS variable was near significance; F = 3.481, *p* = 0.066). The perception of future risk of DEN case introduction at the departmental level (Tables 2 and 3, scenario 1) was best explained by a minimal linear model with birth year, infectiology specialization by the respondents and the interaction term between these two factors as explanatory variables. Concerning the perception of the current risk of DEN case introduction at the national level, two explanatory variables were retained in minimal models (R^2^ = 0.125, AIC = 236.728): infectiology specialization by the respondents (F = 6.125, *p* < 0.05) and longitude (F = 4.351, *p* < 0.05); however, these two variables were close to the 5% confidence interval. For the perception of the current risk of DEN case introduction at the departmental level, the stepwise regression procedure (R^2^ = 0.134, AIC = 257.843) retained the year of medical degree (F = 3.982, *p* = 0.050) and the presence of an international airport (F = 7.496, *p* < 0.005). Finally, for the perception of the future risk of DEN case introduction nationwide, only the NPS (F = 7.638, *p* < 0.01) was retained in the final analysis (R^2^ = 0.095, AIC = 277.983). Over all, professional experience, training and NPS awareness were very important variables for explaining the perception of the current and future risk of DEN case introduction.

**Table 3:** Analysis of variance (based on a Type-III error) to explain the perception of the future risk of dengue case introduction at the department scale after a stepwise backward minimal model using a GLM procedure; *n* = 78 respondents, *R*^2^ = 0.208, *AIC* = 284.467

Source	Mean Squares	*df*	*F*-ratio	*p*-value
Birth year	40.730	1	14.323	< 0.0001
Infectiology specialty	17.218	1	6.055	< 0.05
Birth year × Infectiology specialty	17.042	1	5.993	< 0.05
Error		74	2.844	

#### Autochthonous case situation

At the departmental level, the presence of an international airport and the presence of identified autochthonous human cases of arboviral infection (DEN and/or CHIK) in previous years were the best explanatory variables for the perception of current Zika autochthonous case risk (Tables 2 and 4, scenario 2). Similarly, the models obtained for DEN and CHIK highlighted the presence of an international airport and of tiger mosquitoes as the best explanatory variables for DEN (the NPS variable was near significance, *p* = 0.05) and for CHIK, respectively. At the national level, professional experience in tropical regions and an additional training specialty were overall good predictors of current arboviral disease risk perception. No conclusive or significant results were obtained for the future perception of risk for DEN, CHIK and ZIKA sporadic cases at the department and national levels. Globally, for the autochthonous case scenarios, environmental and geographical independent variables were, for the first time, good predictors at the departmental, but not at the national, level, where professional training and tropical experience were, again, the best explanatory parameters.

**Table 4:** Analysis of variance (based on a Type-III error) to explain the perception of the current risk of Zika autochthonous cases at the department scale after a stepwise backward minimal model using a GLM procedure; *n* = 68 respondents, *R*^2^ = 0.171, *AIC* = 296.256. Autochthonous cases refer to the identification of dengue and/or chikungunya autochthonous cases in the previous year in the respondent’s department

Source	Mean Squares	*df*	*F*-ratio	*p*-value
International airport	41.183	1	9.530	< 0.01
Autochthonous cases	31.814	1	7.362	< 0.01
Error		65	3.726	

#### Epidemic situation

For the epidemic scenario, at the national and department levels, independent variables, such as the respondent’s year of medical degree, birth year, additional specialty and experience in tropical regions, were important parameters for explaining their current and future epidemic risk perception. Using GLMs, the specialty degree and the experience in tropical regions were significant explanatory parameters. This was particularly true for the perception of the current and future DEN epidemic risk. The current DEN epidemic risk perception (Tables 2 and 5, scenario 3) was best explained by the respondent’s training, tropical experience and their two-way interaction terms with the year of medical degree. Concerning the perception of the current and future CHIK and ZIKA epidemic risk, at the national and department levels, no conclusive result was obtained using the null and minimal multivariate models.

**Table 5:** Analysis of variance (based on a Type-III error) to explain the perception of the present dengue epidemic risk nation-wide after a stepwise backward minimal model using a GLM procedure; *n* = 38 respondents, *R*^2^ = 0.547, *AIC* = 156.420. The two independent variables (i.e.*,* professional experience in tropical regions and travel or tropical medicine specialty) are indicated by [1] and [2], respectively, in the interaction terms

Source	Mean Squares	*df*	*F*-ratio	*p*-value
Professional experience in tropical regions [1]	11.235	4	4.180	< 0.01
Travel or tropical medicine specialty [2]	12.766	1	4.750	< 0.05
[1] × year of medical degree	11.307	4	4.207	< 0.01
[2] × year of medical degree	12.747	1	4.743	< 0.05
Error		27	2.668	

## Discussion

### No studies on the arboviral disease risk perception by health professionals

This is the first study on the perception of the present and future risk of vector-borne diseases (i.e., dengue, chikungunya and Zika), in a Western European country, based on an electronic survey completed by French ID physicians in 2016. Many works have focused on disease risk perception in the general population, notably in case of new emerging infectious diseases such as DEN and CHIK [33–36], H1N1pdm flu [37–39] and H5N1 avian flu [40–42]; others have focused on general practitioners in France [43–45], both general population and practitioners [46], French pharmacists [47] or risk perception in Europe and other countries worldwide [48–50]. The appearance of various emerging infectious diseases during the last two decades (e.g., chikungunya, SARS-CoV, MERS-CoV, Ebola virus, Zika), as well as that of antibiotic-resistant bacteria, has stimulated research on risk perception in the general public and policy-makers [51–55]. Information delivered by the media has exacerbated the general feeling, in the public and in national and regional deciders, of the importance of being able to have rapid access to clear information about disease propagation conditions, and to deliver reassuring statements to the population [56, 57]. Conversely, studies on perception risk in the different categories of health professionals, particularly those who directly deal with such diseases and infected patients, are today still very rare, or even absent.

### An exploratory study

This was an exploratory study with several important limitations. First, the percentage of responders was small (11.7%). Second, it is possible that the few who responded were more concerned by vector-borne diseases, and this could have introduced a selection bias into our study. Third, we could not compare the responders with all those on the SPILF Discussion List (*n* = 685) due to missing data, thus preventing any correction for non-responses. This strongly affected the possibility of generalizing our results to the whole community of ID specialists. Nevertheless, we think that these preliminary findings are promising and should stimulate further studies on risk perception within this community.

### Environmental, geographical and epidemiological parameters are less influential than expected in explaining disease risk perception in health professionals

Unexpectedly, the many different statistical models used in this study indicated that environmental, geographical and epidemiological explanatory parameters were not as important as professional training, tropical work experience and NPS awareness in explaining present and future disease risk perception in French ID physicians.

Strangely, the presence of tiger mosquitoes in different departments in the south and southwest of France was not retained as an explanatory variable in minimal models, in most cases. Indeed, the perception of arboviral disease risk was, on average, no higher among respondents working in a hospital located in a department colonized by tiger mosquitoes than among those working in the north of France, where this vector species is absent. The variable “presence of tiger mosquitoes” was significant for the analysis of risk perception only at the departmental level and for CHIK, particularly when studying the difference in risk perception between physicians working in the Hérault department where CHIK cases occurred in 2014 [58] and those from all the other departments. The presence of human autochthonous cases of DEN and CHIK during the previous years in different French departments (see Additional file 1: Appendix III) was an explanatory variable for disease risk perception at the department level, but only for Zika (see below). Moreover, like for the presence of tiger mosquitoes, latitude was never an explanatory variable in regression models, thus indicating that disease risk perception by this category of health professionals is not sensitive to a north-south gradient. Conversely, the perception of the risk of DEN, CHIK and ZIKA epidemic spread was higher among respondents working in departments located on or close to the Mediterranean or Gulf of Biscay coasts than among those working in departments close to Germany or Switzerland. Surprisingly, the presence of an international airport in the respondent’s department was not an important explanatory variable for the disease risk perception, except for DEN and ZIKA at the departmental level. This could be explained by the fact that our questionnaire coincided with the onset of the ZIKA epidemic in Latin America [59, 60] and information on ZIKA risk was at that time delivered by the French health authorities to health professionals and travelers on the occasion of the Olympic Games in Brazil, August 2016 [61]. For the year 2016, the World Health Organization also identified major DEN outbreaks in different parts of the world (South America, Philippines, Malaysia, Salomon Islands, Burkina Faso). This could also explain the effect of the ‘presence of human autochthonous cases’ variable on risk perception for ZIKA outbreaks locally. However, this does not explain why the presence of an international airport was not retained as an explanatory parameter for the risk of DEN, CHIK or ZIKA case introduction from abroad.

### Professional training, tropical experience and NPS awareness influence disease risk perception by health professionals

Most of the linear models indicated that professional training components (medical school cursus, practical experience and current medicine activities) were often significant variables for explaining disease risk perception in health professionals. Having an infectiology or additional training specialty (e.g., travel medicine, epidemiology) strongly influenced their answers in minimizing their perception of arboviral disease risk. This was particularly true for the estimation of imported case risk at the departmental level, and the perception of autochthonous case and epidemic risk at the national level. Moreover, professional experience in tropical regions was an important parameter for explaining perception of arboviral disease risk in mainland France, with generally a lower level of risk perception for respondents with tropical healthcare experience. Many French practitioners traditionally spend time in French overseas territories and developing countries during and after their medical studies. NPS awareness in mainland France was also an important parameter, notably for the imported case scenarios at the departmental and national levels. Undoubtedly, knowledge of the information delivered by the NPS on local disease surveillance and diagnostic practices made respondents more aware of the real situation and lowered the perception of arboviral disease risk compared with ID physicians not aware of the plan. Thus, NPS awareness tended to make respondents more confident about their perception of risk and homogenized the questionnaire responses towards lower risk levels.

### Age and year of medical degree may interact with training components

In some linear models (Table 2), the respondent’s birth year and year of medical degree and their two-way interaction terms with infectiology specialization or with NPS awareness also were important explanatory factors for disease risk perceptions. In particular, the year of medical degree was retained in the regressive models for the perception of imported case risk and, to a lesser extent, of the epidemic risk at both departmental and national levels. The importance of the birth year and year of medical degree suggests that ID physicians of different graduating classes could have received different specialized training on arboviral diseases, with older doctors giving generally lower scores. Alternatively, the younger generations of practitioners are more sensitive to emerging threats due to the recurrence of these events in the last 2 to 3 decades and their significant media coverage. In addition, the two interaction terms birth year×infectiology specialization and birth year×NPS indicated that health professionals without infectiology specialization and who were born in or after 1972 tended to give higher scores (*p* < 0.0001) than the rest of the responders, and that those without NPS awareness and born in or after 1972 tended to give lower scores (*p* < 0.0001). Overall, this suggests that the initial university training strongly impacts current training and professional awareness on risk perception.

### Risk perception is low for sporadic cases but high for epidemics

The dispersion of the values for future sporadic cases (Figs. 1a and 2) suggests that although respondents perceived the current risk of arboviral diseases in mainland France as very low, they imagined all plausible scenarios for future sporadic case risk (from very low to high). Moreover, for higher perception values of current sporadic case risk, the values for future risk tended to converge towards low to medium estimates for the three arboviral diseases and the two scales (see Fig. 1). Nevertheless, we are conscious that a considerable reluctance to extrapolate about the future may exist when “nothing or near nothing” can be perceived today. On the other hand, the future disease risk perception values (Fig. 1b) tended to increase with the increase of the present risk perception values. Two clear patterns of arboviral disease risk perception in mainland France appeared in this study: i) respondents tended to weigh down the future risk of DEN, CHIK and ZIKA sporadic cases in a context of major uncertainty; and ii) they estimated a high level of future epidemic risk. These differences could be explained by the fact that specialists consider themselves and the national authorities effective in controlling the appearance and spread of sporadic disease cases, whereas they see as more limited their capacities to control an epidemic. In addition, for the epidemic scenario, the three diseases were pooled together. Consequently, the responders gave a global response, but were not able to qualify the type of risk: an epidemic could happen in the future whatever its etiological origin.

Interestingly, several recent models on risk of ZIKA outbreaks in the US, based on vector ecology, have suggested disease spread outside of the southernmost counties, a prediction that is inconsistent with actual observations of the ZIKA epidemic on the continent thus far [30, 33]. The findings that we present here are consistent, and would tend to indicate that ID physician perception and its variability (age of training, tropical experience…) of emerging arboviral disease threats may be an important component to be considered in regional and global health security.

## Conclusion

In conclusion, our estimates highlight that the risk of arboviral diseases’ development and spread into mainland France is seen by health professionals as being low overall, which probably represents a good approximation of reality. However, the introduction of dengue, chikungunya and Zika infected cases imported from epidemic and endemic areas will increase with human transportation and displacement into those regions, and our main recommendations are to prioritize communication with citizens and training among health professionals as the best ramparts against these potential infections and the views that people develop around them.

## Additional file

Additional file 1: **Appendix I** Questionnaire and variables added for the study. **Appendix II** Summary of the respondents’ main features. **Appendix III** Summary of the number of confirmed dengue, chikungunya and Zika autochthonous cases in metropolitan France between 2010 and 2019 (date of June 14th, 2019). (DOCX 22 kb)

## Data Availability

The datasets used and/or analysed during the current study are available from the corresponding author on reasonable request.

## Abbreviations

AIC: Akaike Information Criterion
ANOVA: Analysis of variance
CHIK: Chikungunya
CV: Coefficient of variation
DEN: Dengue
GLM: General linear model
GLMM: Generalized Linear Model(s)
ID: Infectious diseases
LOWESS: Locally-weighted scatterplot smoothing
MERS-CoV: Middle East respiratory syndrome coronavirus
NPS: National plan against the spread
REACTing: Research and ACTion targeting emerging infectious diseases
SPILF: French Infectious Diseases Society
SRAS-CoV: Severe acute respiratory syndrome coronavirus
ZIKA: Zika
