# Supplementary material for: Low to medium-low risk perception for dengue, chikungunya and Zika outbreaks by infectious diseases physicians in France, Western Europe
Source: BMC Public Health. 2019 Jul 31;19:1014. doi: 10.1186/s12889-019-7317-9 (PMC6889449; doi:10.1186/s12889-019-7317-9)
Supplement: Supplementary file 1 — Appendix I Questionnaire and variables added for the study. Appendix II Summary of the respondents’ main features. Appendix III Summary of the number of confirmed dengue, chikungunya and Zika autochthonous cases in metropolitan France between 2010 and 2019 (date of June 14th, 2019). (DOCX 22 kb) [file 12889_2019_7317_MOESM1_ESM.docx]

**APPENDIX**

Appendix I – Questionnaire and variables added for the study

| No. | QUESTIONNAIRE |
| --- | --- |
| 1 | Are you…? □ a woman □ a man |
| 2 | When were you born? |
| 3 | In what year did you receive you Doctor of Medicine degree? |
| 4 | What was your initial training specialty? □ Internal medicine □ General medicine □ Public Health □ Others: |
| 5 | Have you obtained one or more additional university diploma(s) in the following specialties? □ Travel and tropical medicine □ Infectiology □ Epidemiology  □ Others: |
| 6 | If yes, in what year? |
| 7 | In what professional structure do you work mainly? Please, indicate the name of the institution, its postal code, and since when you have been working there (for example: infectious diseases service of the university hospital of Montpellier, 34000, since September 2010). |
| 8 | Do you work in other(s) institution(s)? □ Yes □ No |
| 9 | If yes, please indicate the name of the institution(s), postal code and since when you have been working there. |
| 10 | Did you work in tropical regions? □ Yes □ No |
| 11 | If yes, please indicate in which region(s) / country(ies) and periods (e.g., French Guyana, from September 2010 to August 2011; Madagascar, from January to June 2004…). |
| 12 | Are you a member of the French Infectious Diseases Society (SPILF)? □ Yes □ No |
| 13 | Do you take part in scientific meetings and congress (in)directly organized by SPILF ? □ Yes □ No □ I do not know |
| 14 | Currently, what is your estimation of the total number of dengue imported cases nation-wide? (from 0: no imported case, to 10: high number of imported cases). |
| 15 | Currently, what is your estimation of the total number of chikungunya imported cases nation-wide? (from 0: no imported case, to 10: high number of imported cases). |
| 16 | Currently, what is your estimation of the total number of dengue imported cases in your department? (from 0: no imported case, to 10: high number of imported cases). |
| 17 | Currently, what is your estimation of the total number of chikungunya imported cases in your department? (from 0: no imported case, to 10: high number of imported cases). |
| 18 | And in 10 years, how do you estimate the number of imported cases of dengue nation-wide? (from 0: no imported case, to 10: high number of imported cases). |
| 19 | In 10 years, how do you estimate the number of the imported cases of chikungunya nation-wide? (from 0: no imported case, to 10: high number of imported cases). |
| 20 | In 10 years, how do you estimate the number of imported cases of dengue in your department? (from 0: no imported case, to 10: high number of imported cases). |
| 21 | In 10 years, how do you estimate the number of the imported cases of chikungunya in your department? (from 0: no imported case, to 10: high number of imported cases). |
| 22 | In 2015, the French High Council for Public Health defined as “real” the risk of introduction and dissemination of the Zika in mainland France. On the basis of the current international epidemiological situation, do you agree with this statement? □ Totally agree □ Mostly agree □ Mostly disagree □ Totally not agree |
| 23 | Currently, what is your estimation of the risk of dengue sporadic cases nation-wide? (from 0: no risk, to 10: high risk). |
| 24 | Currently, what is your estimation of the risk of chikungunya sporadic cases nation-wide? (from 0: no risk, to 10: high risk). |
| 25 | Currently, what is your estimation of the risk of Zika sporadic cases nation-wide? (from 0: no risk, to 10: high risk). |
| 26 | Currently, what is your estimation of the risk of dengue sporadic cases in your department? (from 0: no risk, to 10: high risk). |
| 27 | Currently, what is your estimation of the risk of chikungunya sporadic cases in your department? (from 0: no risk, to 10: high risk). |
| 28 | Currently, what is your estimation of the risk of Zika sporadic cases in your department? (from 0: no risk, to 10: high risk). |
| 29 | Currently, what is your estimation of the risk of a dengue epidemic nation-wide? (from 0: no risk, to10: high risk). |
| 30 | Currently, what is your estimation of the risk of a chikungunya epidemic nation-wide? (from 0: no risk, to 10: high risk). |
| 31 | Currently, what is your estimation of the risk of a Zika epidemic nation-wide? (from 0: no risk, to 10: high risk). |
| 32 | Currently, what is your estimation of the risk of a dengue epidemic in your department? (from 0: no risk, to 10: high risk). |
| 33 | Currently, what is your estimation of the risk of a chikungunya epidemic in your department? (from 0: no risk, to 10: high risk). |
| 34 | Currently, what is your estimation of the risk of a Zika epidemic in your department? (from 0: no risk, to 10: high risk). |
| 35 | For the 10 next years, what is your estimation of the risk of dengue, chikungunya and Zika sporadic cases nation-wide? (from 0: no risk, to 10: high risk). |
| 36 | For the 10 next years, what is your estimation of the risk of dengue, chikungunya and Zika sporadic cases in your department? (from 0: no risk, to 10: high risk). |
| 37 | For the 10 next years, what is your estimation of the risk of an epidemic of dengue, chikungunya or Zika nation-wide? (With 0: no risk and 10: a real high risk). |
| 38 | For the 10 next years, what is your estimation of the risk of an epidemic of dengue, chikungunya or Zika in your department? (from 0: no risk, to 10: high risk). |
| 39 | Overall, what are your feelings about the risk of dengue, chikungunya or Zika sporadic cases nation-wide? (from 0: no risk, to 10: high risk). |
| 40 | Overall, what are your feelings about the risk of an epidemic of dengue, chikungunya or Zika nation-wide? (from 0: no risk, to 10: high risk). |
| 41 | What is your estimation of the clinical consequences of a dengue epidemic nation-wide? (from 0: no consequences, to 10: severe consequences). |
| 42 | What is your estimation of the socioeconomic impact of a dengue epidemic nation-wide? (from 0: no impact, to 10: high impact). |
| 43 | What is your estimation of the clinical consequences of a chikungunya epidemic nation-wide? (from 0: no consequences, to 10: severe consequences). |
| 44 | What is your estimation of the socioeconomic impact of a chikungunya epidemic nation-wide? (from 0: no impact, to 10: high impact). |
| 45 | What is your estimation of the clinical consequences of a Zika epidemic nation-wide? (from 0: no consequences, to 10: severe consequences). |
| 46 | What is your estimation of the socioeconomic impact of a Zika epidemic nation-wide? (from 0: no impact, to 10: high impact). |
| 47 | Each year, do you receive recommendations on dengue management? □ Yes □ No □ I do not know |
| 48 | Each year, do you receive recommendations on chikungunya management? □ Yes □ No □ I do not know |
| 49 | Each year, do you receive recommendations on Zika management? □ Yes □ No □ I do not know |
| 50 | If yes, from which institution(s) do they come from? □ French Regional Health Agency (ARS) □ The National Institute for Public Health Surveillance (InVS) □ Health Ministry □ French national institute for prevention and health education (INPES) □ The national center for vectors expertise (CNEV) □ I do not know □ Other: |
| 51 | How do you receive these recommendations? (several possible answers) □ By post □ By email □ By telephone □ I do not know  □ Other: |
| 52 | Do these recommendations sound relevant and appropriate? □ Yes, absolutely □ Yes, almost □ Not really □ Not at all □ I do not know |
| 53 | Do you think that communication to health professionals on patients’ management recommendations should be improved? □ Yes □ No □ I do not know |
| 54 | If yes, what type(s) of message(s) and mean(s) of communication would you recommend? |
| 55 | Do you signal suspected case to the French Regional Health Agency (ARS)? □ Yes □ No □ I do not know |
| 56 | If yes, how? □ By telephone □ By fax □ By email □ By post □ I do not know □ Other: |
| 57 | Do you think you need to declare to the health authorities : □ Suspected imported cases □ Suspected autochthonous cases □ Confirmed imported cases □ Confirmed autochthonous cases □ I do not know |
| 58 | Each year, do you get patient-targeted prevention recommendations about dengue, chikungunya and Zika (by official mails, documents with directives, information messages)? |
| 59 | If yes, from which institution(s) do they come from? □ French Regional Health Agency (ARS) □ The National Institute for Public Health Surveillance (InVS)  □ Health Ministry □ French national institute for prevention and health education (INPES) □ The national center for vectors expertise (CNEV) □ Medical and learned journals □ I do not know □ Other: |
| 60 | How do you receive these recommendations? (several possible answers) □ By post □ By email □ By telephone □ I do not know  □ Other: |
| 61 | Do these recommendations sound relevant and appropriate? □ Yes, absolutely □ Yes, almost □ Not really □ Not at all □ I do not know |
| 62 | Do you make these prevention recommendations about dengue, chikungunya and Zika available to your patients? □ Yes, absolutely □ Yes, almost □ Not really □ Not at all □ I do not know |
| 63 | If no, please explain why |
| 64 | If yes, how do you make them available to your patients? (several possible answers) □ Orally, during consultations / hospitalizations □ Posters in the waiting room  □ Documents given to the patients □ Participation in public meetings □ I do not know □ Other: |
| 65 | Do you think that communication (about prevention) to the general public in mainland France should be improved? □ Yes □ No □ I do not know |
| 66 | If yes, what type(s) of message(s) and mean(s) of communication would you recommend? |
| 67 | Have you ever heard about the National plan against the spread of dengue and chikungunya? □ Yes □ No □ I do not know |
| 68 | If yes, how do you judge this plan? □ Very satisfactory □ Quite satisfactory □ Not very satisfactory □ Not satisfactory at all □ I do not know |
| 69 | Do you think that a National plan against the spread of arboviruses in mainland France would be more appropriate than a national plan dedicated to specific viruses (dengue, chikungunya)? □ Yes □ No □ I do not know |
| 70 | Is your department colonized by *Aedes albopictus* mosquitoes (or “tiger mosquitoes”)? □ Yes □ No □ I do not know |
| 71 | Space for comments |
| 72 | If you wish to be informed of the survey results, please enter your email address. |
| **ADDED VARIABLES** | |
| 73 | Latitude (based on the address of the professional structure declared) |
| 74 | Longitude (based on the address of the professional structure declared) |
| 75 | International airport in the relevant department |
| 76 | *Aedes albopictus* implantation in the relevant department |
| 77 | Number of inhabitants in the city of the declared professional structure |
| 78 | Confirmed autochthonous cases identified in the relevant department scale |
| **INITIAL QUESTIONS NOT INCLUDED IN THE ANALYSIS** | |
| / | To which medical laboratory do you send biological samples? (Please indicate the name of the laboratory and city). |
| / | How long does it take to receive the results? |
| / | In 2015, how many suspected / confirmed dengue cases do you think you saw in consultation? |
| / | In 2014, how many suspected / confirmed dengue cases do you think you saw in consultation? |
| / | In 2013, how many suspected / confirmed dengue cases do you think you saw in consultation? |
| / | In 2015, how many suspected / confirmed chikungunya cases do you think you have seen in consultation? |
| / | In 2014, how many suspected / confirmed chikungunya cases do you think you saw in consultation? |
| / | In 2013, how many suspected / confirmed chikungunya cases do you think you saw in consultation? |
| / | In 2015, how many suspected / confirmed dengue cases were hospitalized in your service? |
| / | In 2014, how many suspected / confirmed dengue cases were hospitalized in your service? |
| / | In 2013, how many suspected / confirmed dengue cases were hospitalized in your service? |
| / | In 2015, how many suspected / confirmed chikungunya cases were hospitalized in your service? |
| / | In 2014, how many suspected / confirmed chikungunya cases were hospitalized in your service? |
| / | In 2013, how many suspected / confirmed chikungunya cases were hospitalized in your service? |

Appendix II – Summary of the respondents’ main features

| VARIABLE | MODALITY | NUMBER PER MODALITY (N=) | MISSING NUMBER | PERCENTAGE PER MODALITY (%) |
| --- | --- | --- | --- | --- |
| Sex | Women | 30 | 0 | 37.5 |
|  | Men | 50 |  | 62.5 |
| Age class | ≤ 45-year-old | 40 | 0 | 50 |
|  | > 45-year-old | 40 |  | 50 |
| Year of medical degree | > 2002 | 39 | 2 | 49 |
|  | ≤ 2002 | 39 |  | 49 |
| Initial training specialty | Internal medicine | 30 | 0 | 38 |
|  | General medicine | 28 |  | 36 |
|  | Public Health | 8 |  | 10 |
|  | Others | 12 |  | 15 |
| Additional specialty (several possible answers) | Travel and tropical medicine | 41 |  | 33 |
|  | Infectiology | 54 |  | 44 |
|  | Epidemiology | 20 |  | 16 |
|  | Others | 8 |  | 7 |
| Professional experience in a tropical region | Yes | 41 | 0 | 51 |
|  | No | 39 |  | 49 |
| In which tropical region(s)? | Africa | 15 | 0 | 37 |
|  | America | 5 |  | 6 |
|  | Caribbean | 4 |  | 5 |
|  | South Asia |  |  |  |
|  | Indian Ocean | 1 |  | 1 |
|  | Oceania |  |  |  |
|  | Multiple choices | 16 |  | 20 |
| *Aedes albopictus* establishment in the responder’s department | Yes | 29 | 5 | 36 |
|  | No | 46 |  | 57.5 |
| Autochthonous cases in the responder’s department | Yes | 10 | 4 | 12.5 |
|  | No | 66 |  | 82.5 |
| International airport in the responder’s department | Yes | 44 | 6 | 55 |
|  | No | 30 |  | 37.5 |
| SPILF member | Yes | 48 | 0 | 60 |
|  | No | 32 |  | 40 |

Appendix III – Summary of the number of confirmed dengue, chikungunya and Zika autochthonous cases in metropolitan France between 2010 and 2019 (date of June 14th, 2019). * indicates a sexual transmission identified. See at: <http://invs.santepubliquefrance.fr/Dossiers-thematiques/Maladies-infectieuses/Maladies-a-transmission-vectorielle/Dengue/Donnees-epidemiologiques>

| YEAR | NUMBER OF AUTOCHTONOUS CASES PER DEPARTMENT BETWEEN 2010 AND 2017 | | | | | |
| --- | --- | --- | --- | --- | --- | --- |
|  | Dengue | Department | chikungunya | Department | Zika | Department |
| 2010 | 2 | *Alpes-Maritimes* | 2 | *Var* | 0 | . |
| 2011 | 0 | . | 0 | . | 0 | . |
| 2012 | 0 | . | 0 | . | 0 | . |
| 2013 | 1 | *Bouches-du-Rhône* | 0 | . | 0 | . |
| 2014 | 4 | *Var; Bouches-du-Rhône* | 11 | *Hérault* | 0 | . |
| 2015 | 6 | *Gard* | 0 | . | 0 | . |
| 2016 | 0 | . | 0 | . | 0 | . |
| 2017 | 0 | . | 17 | *Var* | 1* | *Alpes-Maritimes* |
| 2018 | 8 *Alpes-*  *Maritimes,*  *Gard, Hérault* | | 0 . | | 0 . | |
| 2019 | 0 . | | 0 . | | 0 . | |
| In total per disease | DEN = 21 | | CHIK = 30 | | ZIKA = 1 | |
| In total all diseases mixed | 52 | | | | | |
